# Mental health of adolescents before and after the death of a parent or sibling

**DOI:** 10.1007/s00787-015-0695-3

**Published:** 2015-03-19

**Authors:** Yvonne Stikkelbroek, Denise H.M. Bodden, Ellen Reitz, Wilma A.M. Vollebergh, Anneloes L. van Baar

**Affiliations:** Child and Adolescent Studies, Utrecht University, PO Box 80.140, 3508 TC Utrecht, The Netherlands

**Keywords:** Longitudinal, Parental bereavement, Sibling bereavement, Mental disorders, Prospective study

## Abstract

The death of a parent or sibling (family bereavement) is associated with mental health problems in approximately, 25 % of the affected children. However, it is still unknown whether mental health problems of family-bereaved adolescents are predicted by pre-existing mental health problems, pre-loss family functioning, or multiple bereavements. In this study, a prospective longitudinal assessment of change in mental health following bereavement was done in a large representative sample from the ‘Tracking Adolescents Individual Lives Survey’ (TRAILS). This is a four-wave prospective cohort study of Dutch adolescents (*n* = 2230) of whom 131 (5.9 %) had experienced family bereavement at the last wave (T4). Family-bereaved adolescents reported more internalizing problems, within 2 years after family bereavement, compared to the non-bereaved peers, while taking into account the level of internalizing problems before the bereavement. A clinically relevant finding was that 22 % new cases were found in family-bereaved, in comparison to 5.5 % new cases in non-bereaved. Low SES predicted more internalizing problems in family-bereaved but not in non-bereaved adolescents. Family functioning, reported by the adolescent, did not predict mental health problems within 2 years. Multiple family bereavements predicted fewer externalizing problems. In conclusion, internalizing problems increase in adolescents after family bereavement in comparison to non-bereaved and these can be predicted by pre-loss factors. Awareness among professionals regarding the risks for aggravation of mental health problems after family loss is needed.

**Electronic supplementary material:**

The online version of this article (doi:10.1007/s00787-015-0695-3) contains supplementary material, which is available to authorized users.

## Introduction

The death of a parent or sibling during adolescence is a tragic, irreversible loss, which leads to elevated levels of psychological distress [1–6]. The prevalence of the loss of a parent or sibling varies between 1 and 5 % in previous studies [3, 7]. The majority of bereaved adolescents exhibit acute grief reactions, sleep problems, anger, irritability, and behavioural problems [6] and lower self-esteem [8]. These grief reactions can give rise to serious concerns in parent(s) and teachers about the psychological adjustment of the adolescents, although the reactions can still be normal since 75–80 % of the children do not develop mental health problems after the death of a parent [1, 9–12] or sibling [1, 12]. Psychological adjustment after parental bereavement is most commonly characterized by depressive symptoms [10]. Parentally bereaved adolescents are at risk for developing internalizing disorders [8], including major depressive episodes [14]. Furthermore, children who lose a parent or a sibling are at risk for the same mental health problems [3, 12].

Adolescents who overcome bereavement without developing serious mental health problems may have certain protective factors in common [13]. However, there is a lack of systematic attention to protective and risk factors and moderation of psychological adjustment after family bereavement [10]. For professionals working within schools, hospitals and mental health care institutions, knowledge about protective and risk factors present before family bereavement may contribute to identifying children who are at risk for developing (more) mental health problems after bereavement. Early detection may prevent further aggravation of mental health problems or prevent unnecessary psychological treatment and psychiatric stigmatization.

Luecken [11] proposed a comprehensive model of pathways linking early parental death to mental and physical health problems including risk factors. Risk factors for developing mental health problems can be divided in risk factors pre- and post-bereavement. Post-bereavement risk factors have already been identified, namely poorer quality of parenting, worse quality of the parent–child relationship, caregiver mental health problems, subsequent negative life events, low social economic status (SES) and low self-system beliefs, including self-esteem, self-efficacy and social relatedness [10, 11]. However, risk factors may already be present before the loss occurs and may influence psychological adjustment after the loss. According to Dowdney [10] mental health problems of the adolescent before bereavement may constitute an important risk factor because stress caused by the loss can aggravate pre-existing mental health problems. For instance, a depressive disorder is associated with a higher vulnerability to stress [15]. Retrospective studies found that a history of depression [14, 16], sexual abuse [16] and any psychiatric disorder [17] were correlated with depression after parental loss. Furthermore, the presence of mental health problems before bereavement can be associated with the death of a family member. An extensive review of parental cancer showed that a significant number of children developed psychosocial problems during the illness of their parent [18].

In adolescents, gender is a risk factor for increased depressive symptoms in non-bereaved, with girls suffering twice as much as boys [21–23]. Research on gender as a risk factor for depressive problems in family-bereaved adolescents is so far inconclusive [14].

Family bereavement can cause financial hardship (e.g. decrease or loss of income), which may lead to negative life events (e.g. moving house, changing schools and loss of friends) and parenting difficulties [10]. Low socio economic status is in it self associated with more negative life events [9, 24] and parenting difficulties [25] and is therefore associated with greater vulnerability to the effects of family bereavement.

Theoretical and clinical accounts suggest that family functioning, including family organization, cohesion, communication and role differentiation, pre- and post-bereavement is important for the effect of parental bereavement on mental health problems [19]. Family functioning and parenting can be affected by family bereavement in a negative way, for example as a result of parental mental health problems, or in a positive way, when cohesion increases after the loss [10, 19, 20].

Experiencing the death of a parent or sibling renders a child more vulnerable to developing mental health problems in the event of future losses [13]. Experiencing a second family bereavement might have an even greater impact on mental health problems than a first bereavement.

The above-mentioned studies have several limitations. First, most studies in this area do not use a large and representative sample, or a comparison group [26]. Second, research on pre-bereavement family functioning is limited to retrospective accounts of the parents or child about family functioning before bereavement took place, these accounts may be affected by their loss [10]. Third, to our knowledge only one previous study focused on pre-bereavement measurements and prospective analyses of the development of mental health problems. In the current longitudinal study using a large sample (*n* = 2230), the mental health of the adolescents that experienced the death of a parent (*n* = 55) or sibling (*n* = 15) was analysed prospectively.

The present study evaluates the nature and severity of changes in child mental health after bereavement in comparison with a non-bereaved peer population in a large representative sample, hereby taking into account pre-bereavement internalizing and externalizing problems and other potentially confounding variables. First, it is hypothesized that mental health problems, in particular internalizing problems, are more severe in adolescents approximately, 2 years after they experienced death within their family compared to non-bereaved adolescents. Differences in outcomes of family-versus sibling-bereavement will be analysed in an exploratory way. Second, it is hypothesized that more internalizing or externalizing problems before family bereavement are associated with more internalizing or externalizing problems after bereavement compared to the non-bereaved within the same period. Third, by the time adolescents reach the age of 19, those who experienced family bereavement are expected to exhibit more mental health problems (mainly internalizing problems), than their non-bereaved peers. Secondary analyses concern the following predictors in explaining internalizing or externalizing problems after bereavement: internalizing/externalizing problems before bereavement, low family functioning before bereavement, and multiple bereavement.

## Materials and method

### Sample and study design

Subjects were 2230 Dutch participants of a prospective cohort study, the ‘Tracking Adolescents Individual Lives Survey’ (TRAILS). This study was conducted to track the development of mental health from preadolescence into adulthood. The sampling procedure and methods are described in detail in Huisman et al. [27]. Characteristics of the subjects can be found in Table 1. The study has been approved by the Dutch Central Committee on Research Involving Human Subjects. This research was conducted in the northern part of the Netherlands, in an area which supports a variety of economic activities, including (light) industry, services, educational facilities, and agriculture. The participants were recruited from suburban (80 %) and rural (20 %) areas. In total four data collection waves have been completed and these are used in the present study: T1 (2001–2002), T2 (2003–2004), and T3 (2005–2007) and T4 (2008–2010). At all four assessment waves written informed consent was obtained from the adolescents themselves, and for those younger than 18 years, parental consent was also obtained. At T1, parents (mothers, 95.6 %) or guardians were visited at their home by well-trained interviewers and they were asked to fill out self-report questionnaires. The children filled out the questionnaires at school, in the classroom, under the supervision of one or more test assistants (T1, T2, T3). At T4 a web-based questionnaire was used.

**Table 1:** Characteristics of the sample at the four measurements

Total sample	T1	T2	T3	T4	
*n*	2.230	2.149	1.816^a^	1.881^b^	
Mean age (SD)	11.09 (0.56)	13.56 (0.53)	16.27 (0.73)	19.1 (0.60)	
% girls	50.8	51	52.3	52.3	
Response rate (%)	76^c^	96.4^d^	81.4^d^	84.3^d^	

Family bereavement					Total
Deceased persons^e^	70	38	24	23	155
*n* ^f^	52	33	23	23	131
Mean age (SD)	11.14 (0.53)	13.64 (0.54)	16.51 (0.80)	19.24 (0.65)	
% girls	51.7	33.3	69.6	56.5	49.6
Mean SES (SD)^g^	−0.01 (0.21)				

^a^Non-responders at T3 include 2 deceased, 7 who were physically or psychologically unable to participate, 4 who were detained or moved abroad, and 31 untraceable or unreachable participants. Other non-responders refused participation or did not return any information (*n* = 372)

^b^Non-responders at T4 include 5 deceased, 3 who were physically or psychologically unable to participate, and 1 detained participant, 16 untraceable and 43 unreachable participants, and 9 participants who moved abroad. Other non-responders refused participation or did not return any information (*n* = 272)

^c^Of the 2935 eligible children asked to participate at T1

^d^Of the 2230 included children at T1

^e^Occurrence of family-bereavements

^f^Persons who experienced their last family bereavement in this wave

^g^Of the family-bereaved between T1 and T4

At T4, 131 (5.9 %) adolescents had suffered the loss of at least one parent or sibling during their life, and 24 (18 %) of these had lost more than one parent or sibling (see Table 1). In total, 155 family members died before T4. The last bereavement of a parent (*n* = 87; 66 %) or sibling (*n* = 44; 34 %), occurred in 6 % (*n* = 8) within the past 2 months, in 10 % (*n* = 10) between 2 and 12 months previously and in 78 % (*n* = 102) more than 12 months previously, with 6 % (*n* = 8) unknown. A total of 79 adolescents experienced the death of a parent or sibling between T1 and T4. Some family-bereaved did not participate at all in one of the assessments (pre- or post-bereavement) and these were regarded as dropouts (*n* = 9). The reasons for dropout are unknown. The remaining 70 adolescents were included in the analyses.

### Instruments

#### Demographic variables

Gender, age, and SES [28] of the parents were assessed during an interview with a questionnaire at T1 with one of the parents. SES was based on a scale containing educational level (father/mother), occupation (father/mother), and family income. The internal consistency was good (*α* = 0.84) [28].

#### Death within the family

Family bereavement was assessed by asking the adolescents if they had lost a parent or sibling by death, including stepparents, stepbrothers, stepsisters, half-brothers and half-sisters, yes or no.

#### Multiple bereavement within the family

This was assessed by summing the scores on death within the family in the waves prior to the last bereavement. The bereavements prior to the last family-bereavement occurred in wave T1, T2, T3 or T4.

#### Mental health problems

Internalizing and externalizing mental health problems were assessed with the Youth Self Report (YSR) [29] at T1 to T3 and the Adult Self-Report (ASR) [30] at T4. Both questionnaires contain a similar list of emotional and behavioural problems which are rated on a scale of 0 = not true, 1 = somewhat or sometimes true or 2 = very or often true, in the past 6 months. A higher score indicates more symptoms of psychopathology. Scale scores were converted into standardized scores for the YSR internalizing (31 items *M* = 11.27, *α* = 0.87) and externalizing dimensions (32 items *M* = 8.51, *α* = 0.85) and the ASR internalizing (39 items *M* = 9.83, *α* = 0.93) and externalizing dimensions (35 items *M* = 8.01, *α* = 0.89). The pre (loss) score, before bereavement, was obtained by selecting internalizing and externalizing subscale scores of the YSR at the beginning of the period in which the (last) bereavement took place. As post (loss) score, the YSR score on internalizing or externalizing problems at the end of the period in which bereavement took place was used. In the case of multiple bereavements, the last bereavement nearest to T4 was selected as the target event.

#### Family functioning

Family functioning was assessed at the wave prior to the bereavement with a modified version of the General Scale of the McMaster Family Assessment Device (FAD) [31] (12 items *α* = 0.85). Parents could rate how they agreed with statements concerning the functioning of their family on a 4-point scale 1 = totally disagree, 2 = disagree, 3 = agree, 4 = totally agree. A low score on the scale indicates a healthy family climate and a high score represents a dysfunctional family climate (12 items *α* = 0.85). Only the pre (loss) score was used.

### Statistical analyses

The incomplete cases per scale (YSR, ASR, FAD) were imputed to maximize the number of complete cases with corrected item mean imputation (CIM) [32] in which person information as well as scale information was used. Scales larger than five items were not imputed if 50 % or more of the answers were missing and the scale score was not computed. If subjects did not participate in the wave before or after the bereavement they were not included (*n* = 9). Consequently, the data of 70 adolescents who experienced the death of a parent (79 %, *n* = 55) or a sibling (21 %, *n* = 15) during wave 2, 3 or 4 were included in the analyses and treated as one group with pre- and post-assessments at T1, T2, T3 or T4 (see Table 1). None of the bereaved adolescents lost both, a parent and a sibling. We wanted to compare the family-bereaved with the non-bereaved. Since not all three time periods (between four assessments) could be selected due to inter-correlation of those assessments only one time period was selected per non-bereaved individual. This resulted in one time period with two assessments that matched the bereaved group of which only one time period was chosen in which family bereavement took place. Therefore, the non-bereaved adolescents (non-bereaved, *n* = 2099) were randomly assigned to three groups (A, B and C) by SPSS (IBM SPSS Statistics 21.0, 2012) [33]. The assessments of Group A at T1 were included as pre-score and at T2 as post score. The assessments of group B at T2 were included as pre-score and at T3 as post score. The assessments of group C at T3 were included as pre-score at T4 as post score. The aforementioned assessments of Group A, B and C together formed the non-bereaved group with pre- and post-scores of the wave. These scores are referred to as pre (loss) score and post (loss) score.

Paired sample *t* tests were conducted to test changes in internalizing and externalizing problems between pre- and post-bereavement specified for parental bereavement, sibling bereavement, family bereavement and for the non-bereaved.

An independent sample *t* test was conducted to test differences between bereaved and non-bereaved and within-group changes on internalizing and externalizing problems after computing the difference score: post (loss) score minus pre-test score. Effect sizes and pooled Cohen’s *d* were calculated. To establish clinically significant changes in internalizing and in externalizing problems before and after the loss of a family member, the cut-off score for clinical cases, according to the Dutch standard of the YSR and ASR, in internalizing and externalizing problems was used. The change in number of clinical cases pre (loss) and post (loss) was analysed in both the bereaved and non-bereaved sample. An increase in clinical cases was calculated by the number of clinical cases post-loss that were not clinical at pre-loss, divided by the number of nonclinical cases at pre-loss. A Chi-square test was conducted. Changes in family functioning from pre to post-loss were also tested with paired sample *t* tests for the bereaved and non-bereaved.

Hierarchical multiple regression analysis was employed to determine if internalizing and externalizing problems at T4 could be predicted by family bereavement (yes/no) after controlling for gender and SES.

Hierarchical multiple regression analysis were conducted for the family-bereaved between T1 and T4, to determine if functioning [internalizing or externalizing problems, family functioning, number of family bereavements (life time)] prior to bereavement predict internalizing or externalizing problems after bereavement while controlling for gender and SES. This hierarchical multiple regression analysis was repeated once more for the non-bereaved group (without the variable amount of bereavement). Because of the small size of the family-bereaved group, we opted to conduct two separate hierarchical multiple regression analysis for the family-bereaved and the non-bereaved. In the family-bereaved group the number of family bereavements (life time) was added as the last step.

## Results

### Post (loss) mental health and type of bereavement

An independent *t* test of internalizing problems between the family-bereaved and non-bereaved subsample. Internalizing problems in family-bereaved (*M* = 0.08, SD = 0.27) increased significantly in comparison to non-bereaved (*M* = −0.04, SD = 0.22) from pre to post (loss) score over the same period *t* (1168) = −3.97, *p* < 0.001, showing a medium effect (Cohen’s *d* pooled 0.37; 95 % CI 0.13–0.62).

As a more rigorous measure, the clinical cut-off scores for the subscale internalizing problems were used to assess new clinical cases at post (loss) to establish clinically relevant changes. The increase in clinical cases was 22 versus 5.5 %, which is four times higher in family-bereaved than in non-bereaved over the same period. The difference in increase in clinical cases was tested with a Chi-square test and was found to be significant *χ* ^2^ (1) = 16.46, *p* < 0.001, a small effect (Cramers*’V* 0.10, *p* < 0.001).

Within-group analysis showed that internalizing problems increased significantly with a small effect when family bereavement occurred within the past 2 years (see Table 2). In the non-bereaved, a significant decrease with a small effect of internalizing problems was found for this period*, t* (1213) = 6.2, *p* < 0.01. The increase in internalizing problems after sibling bereavement was higher than after parental bereavement, but was not significant.

**Table 2:** Comparison of family-bereaved at T2, T3, T4 (*n* = 70), including sibling bereaved (*n* = 15) and parental bereaved (*n* = 55), and non-bereaved (*n* = 1213 internalizing and *n* = 1222 externalizing problems) on pre- and post-internalizing and externalizing problems

	Internalizing problems						Externalizing problems						New clinical cases^b^	
	Pre		Post		*T*	E.S^a^	Pre		Post		*T*	E.S^a^		
	*M*	SD	*M*	SD			*M*	SD	*M*	SD			Int (%)	Ext (%)
Parental bereavement	0.34	0.23	0.39	0.28	−1.72	0.19	0.31	0.20	0.35	0.24	−1.04	0.18	19	9
Sibling bereavement	0.37	0.28	0.52	0.41	−1.61	0.41	0.26	0.20	0.32	0.19	−0.92	0.31	22	8
Family bereavement	0.34	0.24	0.42	0.31	−2.35*	0.28	0.30	0.20	0.34	0.23	−1.35	0.19	22	9
Non-bereavement	0.33	0.24	0.29	0.25	6.2**	0.16	0.27	0.19	0.28	0.20	−1.04	0.05	5.5	5.7

*M* mean

* *p* < 0.05, ** *p* < 0.01

^a^Effect size: Cohen’s *d* corrected for correlation of pre-test and post-test

^b^Percentage clinical cases at post-test which were not yet clinical case at pre-test

An independent *t* test was conducted to test the changes in externalizing problems between both groups and this showed a significant increase in family-bereaved (*M* = 0.04, SD = 0.25) compared to non-bereaved (*M* = −0.01, SD = 0.19), *t* (1180) = −1.99, *p* < 0.05, with a small effect (Cohen’s *d* pooled 0.12; 95 % CI −0.36 to 0.12). This difference in increase in clinical cases with externalizing problems between family-bereaved and non-bereaved was 2.3 %, which is very small, and not significant *χ* ^2^ (1) = 2.17, *p* > 0.05. Within both groups no differences were found in externalizing problems from pre to post (loss) (see Table 2).

### Post (loss) mental health at age of 19

Two hierarchical multiple regression analyses were conducted separately for internalizing and externalizing problems at T4 to examine the effect of family bereavement during youth (0–19). In the first step, demographics (gender, SES) were added. In the second step, family-bereaved (*M* = 3.03, SD = 0.81) versus non-bereaved (*M* = 3.17, SD = 0.70) was added (see Table 3).

**Table 3:** Mental health problems, internalizing and externalizing problems, in family-bereaved as well as non-bereaved adolescents

Predictor	Post internalizing problems		Post externalizing problems	
	*∆R* ^*2*^	*β*	*∆R* ^*2*^	*β*
Step1	0.04		0.005	
Gender		−0.19**		0.03
SES T1		−0.07**		−0.07**
Step 2	0.003		0.004	
Family-bereaved yes/no		0.05*		0.06**
Total *R* ^2^	0.04		0.009	
*n*	1580		1581	

* *p* < 0.05, ** *p* < 0.01

The results for internalizing problems showed that female gender (*B* = −0.19, SE = 0.01, *p* < 0.001) and low SES (*B* = −0.07, SE = 0.01, *p* < 0.01) significantly contributed to the variance, *F* (2, 1661) = 34.88, *p* < 0.001. Furthermore, family-bereaved versus non-bereaved explained an additional 0.3 % of the variance, *F* (3, 1660) = 25.02, *p* < 0.001, indicating that experiencing family bereavement is associated with more internalizing problems compared to non-bereaved adolescents. All variables explained four, 3 % of the variance of internalizing problems by the age of 19.

Results concerning post (loss) externalizing problems showed approximately the same results as were found for internalizing problems, except that gender was not significant. SES contributed significantly to the variance, *F* (2, 1661) = 4.32, *p* < 0.05. Family-bereaved versus non-bereaved, explained an additional 0.4 % of the variance *F* (3, 1626) = 5.11, *p* < 0.01, (*B* = 0.06, SE = 0.02, *p* < 0.01) indicating that experiencing family bereavement is associated with more externalizing problems than in non-bereaved at T4.

### Control variables

#### Gender and SES

As presented in Table 4, the hierarchical multiple regression analyses of internalizing problems for family-bereaved showed that the first step, gender and SES, explained 21 % (versus 7 % in non-bereaved); a significant part of the variance. SES at T1 was centred and was lower in non-bereaved (*M* = −0.04, SD = 0.80) than in bereaved (*M* = −0.01, SD = 0.21). Low SES predicted internalizing problems in family-bereaved but not in non-bereaved. Being female predicted internalizing problems in both groups. In both groups Gender and SES did not predict externalizing problems.

**Table 4:** Predictors of change in internalizing and externalizing problems in non-bereaved and family-bereaved

Predictor	Post-internalizing problems			Post-externalizing problems	
	*∆R* ^2^	*β*		*∆R* ^2^	*β*
Family-bereaved					
Step1	0.21			0.01	
Gender		−0.31*			0.10
SES T1		−0.35**			0.03
Step 2	0.18			0.06	
Pre-loss internalizing or externalizing problems		0.47**			0.24
Step 3	0.02			0.02	
Pre-loss Family Functioning		0.16			0.14
Step 4	0.03			0.08	
Multiple bereavement		−0.18			−0.28*
Total *R* ^2^	0.43			0.16	
*n*	70			70	
Non-bereaved					
Step 1	0.07			0.01	
Gender		−0.25**			0.05
SES T1		−0.05			−0.06
Step 2	0.30			0.25	
Pre-loss internalizing or externalizing problems		0.56**			0.50**
Step 3	0.01			0.01	
Pre-loss Family Functioning		0.03			0.08**
Total *R* ^2^	0.37			0.27	
*n*	1007			1007	

* *p* < 0.05, ** *p* < 0.01

### Predictors

#### Pre (loss) mental health problems

Adding pre (loss) levels of internalizing problems in step 2 explained an additional 18 % of the variance in family-bereaved but much more in non-bereaved adolescents namely 30 %. The first and second steps together explained 38 % of the variance of internalizing problems in family-bereaved: adjusted *R* ^2^ is *0.*35 and *F* (3, 52) = *10.68, p* < 0.001, (*B* = 0*.67,* SE = 0*.15, p* < 0.001). In the non-bereaved, both steps explained in total 36 % of the variance, with an adjusted *R* ^2^ of *0.36* and *F* (3, 1000) = 144*.25, p* < 0.001 (*B* = 0.55, SE = 0*.03, p* < 0.001).

Pre (loss) externalizing problems in family-bereaved adolescents did not explain variance in externalizing problems after bereavement *F* (3, 52) = 1.26, *p* < 0.30, (*B* = 0.24, SE = 0.16*, p* > 0.05). In the non-bereaved, externalizing problems at pre-test explained an extra 25 % of the variance, *F* (3, 1005) = 113.06, *p* < 0.001, (*B* = 0.50, SE = 0.03, *p* < 0.001).

In sum: Pre (loss) scores on internalizing problems predicted post (loss) scores on internalizing problems in family-bereaved and in the non-bereaved adolescents. Pre (loss) scores on externalizing problems did not predict externalizing problems after bereavement. In the non-bereaved, however, pre (loss) scores on externalizing problems predicted post (loss) scores on externalizing problems.

#### Pre (loss) family functioning

An independent paired sample *t* test showed that family functioning within the family-bereaved did not significantly change after bereavement *t* (41) = 0.34, *p* > 0.05. In the non-bereaved, family functioning became significantly better over the same period *t* (1090) = 2.13, *p* < 0.05. This was a small effect (Cohen’s *d* pooled 0.13; 95 % CI 0.0102–0.25). The pre (loss) score on family functioning was significantly higher in the family-bereaved (*M* = 1.85, SD = 0.37) compared to the non-bereaved (*M* = 1.68, SD = 0.39), *t* (1327) = −3.22, *p* < 0.001).

An independent *t* test was conducted to test the difference in changes in family functioning between the family-bereaved and non-bereaved subsample. The difference score on family functioning, post (loss) score minus pre (loss) score was used. Change in family functioning in family-bereaved (*M* = −0.02, SD = 0.42) was not different compared to non-bereaved (*M* = −0.05, SD = 0.38) from pre- to post (loss) score over the same period *t* (1131) = −0.04, *p* > 0.05.

To examine whether family functioning before bereavement predicted mental health problems after bereavement, family functioning was added in the third step of the regression analysis. The pre (loss) score on family functioning did not predict internalizing problems after controlling for gender, SES and pre (loss) score on internalizing problems in family-bereaved *F* (4, 51) = 8.60*, p* < 0.001, (*B* = 0.16, SE = 0.10*, p* > 0.05), this was also true for non-bereaved adolescents, *F* (4, 1003) = 144.25*, p* < 0.001, (*B* = 0.03, SE = 0.02*, p* > 0.05).

Moreover, pre (loss) score on family functioning was not associated with post (loss) score on externalizing problems in the family-bereaved. In contrast, in the non-bereaved group the pre scores on family functioning predicted post (loss) scores on externalizing problems, indicating that dysfunctional family climate predicted externalizing problems. The additional variance explained was only 1 %*, F* (4, 1003) = 10.68*, p* < 0.001, (*B* = 0.08, SE = 0.01*, p* < 0.01) (see Table 4).

#### Multiple bereavement

Adding multiple bereavement in step 4 of the hierarchical multiple regression analyses (Table 4) showed that the experience of more than one family bereavement (*n* = 24) did not predict internalizing problems after bereavement *F* (5, 55) = 7.64*, p* < 0.001, (*B* = −0.18, SE = 0.05*, p* > 0.05). However, it did predict fewer externalizing problems, while controlling for pre (loss) score externalizing problems: *F* (5, 50) = *1.90, p* < 0.05, (*B* = −0.28, SE = 0.04*, p* < 0.05).

## Discussion

The main aims of the present study were to examine the influence of family bereavement on the mental health of adolescents and to determine which pre-loss factors deteriorate mental health care problems. The main result shows that family bereavement has a clinically significant, medium sized effect on the increase of internalizing problems within 2 years in comparison to non-bereaved adolescents while accounting for pre (loss) internalizing problems. By the age of 19, family-bereaved adolescents experienced significantly more internalizing and externalizing problems than non-bereaved adolescents. The internalizing problems after family bereavement are predicted by the amount of internalizing problems before family bereavement occurred. This was not found for externalizing problems. However, the experience of more than one family bereavement did predict fewer externalizing problems.

### Internalizing problems

Family-bereaved adolescents developed more internalizing problems compared to their non-bereaved peers. Furthermore, the increase in the number of new clinical cases with internalizing problems in family-bereaved was four times as high, namely 22 % in comparison to 5.5 % in the non-bereaved adolescents. These findings are consistent with previous studies which found more internalizing problems in family-bereaved compared to non-bereaved [1, 9–12]. Moreover, by the age of 19 the difference between family-bereaved compared to non-bereaved in internalizing problems was robust. Earlier studies also found that internalizing problems, especially depression, increased within the second year after bereavement [34, 35]. Even after more than 6 years, 13 % of parentally bereaved adolescents who participated in the control group of an intervention study reported internalizing disorders [36]. Although the studies cited above showed an increase in internalizing problems after bereavement, it was still uncertain how much could be accounted for by pre-loss internalizing problems. In our study we were able to show a substantial increase in the amount of internalizing problems in adolescents who did not experience internalizing problems before bereavement. To our knowledge, so far one other study on bereavement has assessed pre-bereavement mental health including generalized anxiety, separation anxiety and depression. The results were mixed, parentally bereaved youth showed an increase in at least one anxiety symptom while controlling for pre-parental loss in comparison to non-bereaved youth but symptoms of depression did not [37]. Research on effects of parental death on adult psychopathology is inconclusive, suggesting that it is unknown if family-bereaved children continue to have more internalizing problems during adulthood [38].

Surprisingly, the increase in internalizing problems after the loss of a sibling seemed substantially higher than after the loss of a parent. This finding was not significant however, probably due to low power of our study in view of its sample size. Despite the non-significant finding, these results are in line with the abovementioned study that also reported an increase in at least one depressive symptom from pre- to post-bereavement in adolescents who experienced losing a sibling or other bereavement, but not in youth losing a parent [37]. It is possible that specific factors are responsible for a higher impact of sibling loss on internalizing problems. For instance, siblings report feeling guilty because they did not die, so called survivor guilt [18]. Also parent’s psychological distress after bereavement plays a role in the development of mental health problems [40] and may be more problematic after losing a child in comparison to losing a spouse [41].

In the present study, we found that low SES predicted internalizing problems in the family-bereaved. When SES was already low before family bereavement, adverse social economic consequences might be more difficult to handle and put an adolescent at an increased risk for internalizing problems [10, 37]. Therefore, special attention should be given to the family-bereaved adolescents growing up in families with low SES. Being female was associated with significant internalizing, but not externalizing, problems in both groups.

We found that an increase in internalizing problems among the family-bereaved as well as in the non-bereaved group is predicted by the amount of pre (loss) internalizing problems, after controlling for gender and SES. A history of internalizing problems before family bereavement puts an adolescent at a higher risk of internalizing problems in the future. This finding is comparable to the finding in a previous study, which showed that a retrospective account of a history of depression before parental bereavement increased the risk for depression in the 9 months following the death of a parent, which in turn increased depression risk between 9 and 21 months [34]. Future research is needed on the selectivity and specificity of these measures by retrospective inquiry for internalizing problems before family bereavement, so professionals within schools, hospitals and mental health care institutions can identify adolescents at risk for internalizing problems.

### Externalizing problems

We hypothesized that externalizing problems would increase after family bereavement compared to non-bereaved youth, but this was not supported by the results. Family-bereaved and non-bereaved did not experience a significant change in the number or extent of externalizing problems from pre- to post (loss) score. The change in clinical cases was 2.3 % more cases in family-bereaved compared to non-bereaved and this was not significant. These findings are not in line with the estimated 10–21 % of bereaved children who develop clinical levels of externalizing disorders [35, 39]. Furthermore, pre (loss) externalizing problems did not predict externalizing problems after family bereavement. This is in contrast to the finding in non-bereaved adolescents, that pre (loss) externalizing problems predicted post (loss) externalizing problems, which explained 25 % of the change in variance. In the non-bereaved, externalizing problems seem stable whereas this is not the case in the family-bereaved. However, we found that by the age of 19, family bereavement between 0 and 19 years significantly predicted more externalizing problems. Changes in externalizing problems after bereavement may be bidirectional. For instance, one study found an increase of externalizing problems because of emotion regulation problems [11]. Other studies found that some adolescents report that externalizing problems diminished after family bereavement because they felt more mature [42] and experienced a greater appreciation of life [43]. For example, incidence of substance abuse and alcohol abuse were found to be lower in adults who were parentally bereaved during childhood [44].

### Multiple bereavements

Almost 30 % of family-bereaved adolescents experienced more than one family bereavement. Surprisingly, this factor predicted less externalizing problems controlling for gender, SES, externalizing problems at pre-loss and family functioning. This finding is not in line with a previous study showing that after experiencing more than one family bereavement the adolescents were more vulnerable to symptoms of grief, such as explosive emotions, acting out, temper tantrums and delinquent activity [13]. A possible explanation for our finding might be, that increasing externalizing behaviour is rather normative in the adolescent years, and is decreased or buffered by the grief and loss that has occurred in these families. Multiple bereavements did also not predict an increase in internalizing problems. As internalizing problems already increased after the first bereavement a further increase could be difficult to detect because of a ceiling effect.

### Family functioning

Family functioning did not change significantly after family bereavement. The difference in change in family functioning in family-bereaved compared to non-bereaved was not significant. Furthermore, poor family functioning only predicted externalizing problems in the non-bereaved, but seemed less relevant, compared to previous levels of internalizing/externalizing problems, because it only explained 1 % of variance of change. In the family-bereaved, family functioning did not predict internalizing or externalizing problems after bereavement. Taking into account that family-bereaved experienced significantly poorer pre (loss) family functioning than the non-bereaved, another explanation needs to be considered. The pre (loss) score on family functioning might already have been affected in some families before bereavement as a result of the impact of the illness that led to the death of the family member. In support of this explanation, an extensive systematic review found that children of cancer patients, who experienced poor family functioning, were at risk for maladjustment [18]. Also, the small sample size in our subgroup may have prevented the finding of predictive factors in the family-bereaved group. The change in family functioning in the family-bereaved group was established in intervention research as a relevant factor in overcoming family bereavement without mental health problems [10, 11].

### Strengths and limitations

Certain limitations of this study need to be addressed. First, some findings were not significant but have to be interpreted with caution because of a potential lack of power due to a small size of the family-bereaved subgroup. Although 131 individuals suffered from family bereavement during their life of whom 70 during adolescence, this is still a small sample if 20–25 % of them are expected to develop mental health problems after family bereavement on the basis of previous research [1, 9–12]. However, 7 % (*n* = 9) of the family-bereaved did not participate in the assessment pre- or post-bereavement, which could have been associated with the burden of family bereavement. Examples are reluctance to answer potentially painful questions during the assessment or having to move house. This suggests that mental health problems after family bereavement might be even more prevalent than found in this study. Another important factor that may contribute to increasing levels of mental health problems following family bereavement is the mental health of the parent pre- to post-bereavement. This could not be taken into account in the present study. The psychological functioning of a parent after the bereavement may have been impaired, resulting in less ability to support the emotional wellbeing of the child. Parental functioning predicts the adjustment of the child [4]. Furthermore, parental depression after bereavement can reduce the capacity for positive parenting [40].

Other characteristics such as the gender of the deceased parent, cause of death and self-esteem also seem important to consider in future studies with larger samples sizes. The cause of death, whether accidental death, illness, suicide or violent death, has been found to be associated with outcome of parental bereavement in terms of mental health [34].

Notwithstanding the limitations, the present study has several strengths. Strengths include the relatively large, representative community sample and longitudinal data. The data on mental health and family functioning were collected before the bereavement occurred, which made prospective analyses possible. It was also possible in our study to adjust for demographic characteristics that might have affected mental health problems. In addition, research on the bereavement process in adolescents thus far was limited to the duration of 2 years after experiencing the death of a parent [26], whereas we were able to examine mental health problems over a period of 8 years.

## Conclusions

Family bereavement puts adolescents at risk for internalizing problems within 2 years and mental health problems by the age of 19, in comparison to non-bereaved peers. The present study identified a history of internalizing symptoms and low social economic status of the family as pre-bereavement predictors of mental health problems after family bereavement. These predictors could be used in further research to identify possibilities for selective prevention after occurrence of a family bereavement in adolescents. Awareness among professionals regarding the risks for aggravation of mental health problems after family loss is needed.

## Electronic supplementary material

Below is the link to the electronic supplementary material.

Supplementary material 1 (DOCX 28 kb)

## References

[1] Dowdney L (2005). Children bereaved by parent or sibling death. Psychiatr.

[2] Fristad MA, Jedel R, Weller RA, Weller EB (1993). Psychosocial functioning in children after the death of a parent. Am J Psych.

[3] Harrison L, Harrington R (2001). Adolescents’ bereavement experiences: prevalence, association with depressive symptoms, and use of services. J Adolesc.

[4] Kalter N, Lohnes KL, Chasin J, Cain AC, Dunning S, Rowan J (2002). The adjustment of parentally bereaved children: I. Factors associated with short-term adjustment. OMEGA J Death Dying.

[5] Siegel K, Karus D, Raveis VH (1996). Adjustment of children facing the death of a parent due to cancer. J Am Acad Child Adolesc Psychiatr.

[6] Silverman PR, Worden JW (1992). Children’s reactions in the early months after the death of a parent. Am J Orthopsychiatr.

[7] Centraal Bureau voor de Statistieken [CBS] (2013) Jaarlijks verliezen ruim 6 duizend minderjarige kinderen een of beide ouders. CBS Webmagazine. http://www.cbs.nl/nl-NL/menu/themas/bevolking/publicaties/artikelen/archief/2013/2013-3869-wm.htm?RefererType=RSSItem&RSSFeedTitle=Bevolking. Accessed 15 Nov 2013

[8] Mack KY (2001). Childhood family disruption and adult well-being: the differential effects of divorce and parental death. Death Stud.

[9] Cerel J, Fristad MA, Verducci J, Weller RA, Weller EB (2006). Childhood bereavement: psychopathology in the 2 years post parental death. J Am Acad Child Adolesc Psychiatr.

[10] Dowdney L (2000). Annotation: childhood bereavement following parental death. J Child Psychol & Psychiatr.

[11] Luecken LJ, Roubinov DS (2012). Pathways to lifespan health following childhood parental death. Soc Pers Psychol Compass.

[12] Worden JW, Davies B, McCown D (1999). Comparing parent loss with sibling loss. Death Stud.

[13] Oltjensbruns KA, Stroebe MS, Hansson RO, Stroebe W, Schut H (2001). Developmental context of childhood: Grief and regrief phenomena. Handbook of bereavement research; consequences, coping and care.

[14] Gray LB, Weller RA, Fristad M, Weller EB (2011). Depression in children and adolescents two months after the death of a parent. J Affect Disord.

[15] Braet C, Vlierberghe LV, Vandevivere E, Theuwis L, Bosmans G (2013). Depression in Early, middle and late adolescence: differential evidence for the cognitive diathesis–stress model. Clin Psychol Psychother.

[16] Melhem NM, Walker M, Moritz G, Brent DA (2008). Antecedents and sequelae of sudden parental death in offspring and surviving caregivers. Arch Pediatr Adolesc Med.

[17] Weller RA, Weller EB, Fristad MA, Bowes JM (1991). Depression in recently bereaved prepubertal children. Am J Psychiatry.

[18] Krattenmacher A, Franziska Kühne F, Ernst J, Bergelt C, Romer G, Möller B (2012). Parental cancer: Factors associated with children’s psychosocial adjustment, a systematic review. J Psychos Res.

[19] Sutcliffe P, Tufnell G, Cornish U (1998). Working with the dying and bereaved: systematic approaches to therapeutic work.

[20] Gilmer MJ, Foster TL, Vannatta K, Barrera M, Davies B, Dietrich MS (2012). Changes in parents after the death of a child from cancer. J Pain Symp Manag.

[21] Lewinsohn PM, Rohde P, Seeley JR (1998). Major depressive disorder in older adolescents: prevalence, risk factors, and clinical implications. Clin Psychol Rev.

[22] Hyde JS, Mezulis AH, Abramson LY (2008). The ABCs of depression: Integrating affective, biological, and cognitive models to explain the emergence of the gender difference in depression. Psychol Rev.

[23] Thapar A, Collishaw S, Pine DS, Thapar AK (2012). Depression in adolescence. Lancet.

[24] Evans GW, English K (2002). The environment of poverty: Multiple stressor exposure, psychophysiological stress, and socio-emotional adjustment. Child Dev.

[25] McLoyd VC (1998). Socio-economic disadvantage and child development. Am Psychol.

[26] Balk DE, Corr CA, Stroebe MS, Hansson RO, Stroebe W, Schut H (2001). Bereavement during adolescence: a review of research. Handbook of bereavement research; consequences, coping and care.

[27] Huisman M, Oldehinkel AJ, de Winter A, Minderaa RB, de Bildt A, Huizink AC (2008). Cohort profile: the Dutch ‘tracking adolescents’ individual lives’ survey’; TRAILS. Int J Epidem.

[28] Vollebergh WA, ten Have M, Dekovic M, Oosterwegel A, Pels T, Veenstra R (2005). Mental health in immigrant children in the Netherlands. Soc Psychiatr Psychiatr Epidem.

[29] Achenbach TM (1991). Manual of the youth self-report and 1991 profile.

[30] Achenbach TM (2003). Rescorla LA: Manual for the ASEBA adult forms and profiles, research center for children, youth & families.

[31] Epstein NB, Baldwin LM, Bishop DS (1983). The McMaster family assessment device*. J Marital Fam Ther.

[32] Raaijmakers Q (1999). Effectiveness of different missing data treatments in surveys with likert-type data: introducing the relative mean substitution approach. Educ Psychol Meas.

[33] IBM Corp Released (2012) IBM SPSS statistics for windows, Version 21.0. IBM Corp, Armonk, New York

[34] Brent D, Melhem N, Donohoe MB, Walker M (2009). The incidence and course of depression in bereaved youth 21 months after the loss of a parent to suicide, accident, or sudden natural death. Am J Psych.

[35] Worden JW, Silverman PR (1996). Parental death and the adjustment of school-age children. OMEGA J Death Dying.

[36] Sandler I, Ayers TS, Tein J, Wolchik SA, Millsap R, Jhoo ST (2010). Six-year follow-up of a preventive intervention for parentally bereaved youths. Arch Pediatr Adolesc Med.

[37] Kaplow JB, Saunders J, Angold A, Costello EJ (2010). Psychiatric symptoms in bereaved versus nonbereaved youth and young adults: a longitudinal epidemiological study. J Am Aca Child Adolesc Psychiatry.

[38] Luecken LJ, Stroebe MS, Hansson RO, Schut H, Stroebe W (2008). Long-term consequences of parental death in childhood: psychological and physiological manifestations. Handbook of bereavement research and practice; advances in theory and intervention.

[39] Lifton RJ (1967) Death in life. New York Random House

[40] Kwok OM, Haine RA, Sandler IN, Ayers TS, Wolchik SA, Tein JY (2005). Positive parenting as a mediator of the relations between parental psychological distress and mental health problems of parentally bereaved children. J Clin Child Adolesc Psychol.

[41] Sanders C. A comparison of adult bereavement in the death of a spouse, child and parent. Omega J Death Dying 10:303–322. doi:10.2190/X565-HW49-CHR0-FYB4

[42] Davies B (1991). Long-term outcomes of adolescent sibling bereavement. J Adolesc Res.

[43] Brewer JD, Sprakes AC (2011). Young people living with parental bereavement: insights from an ethnographic study of a UK childhood bereavement service. Soc Sci Med.

[44] Stikkelbroek Y, Prinzie P, Graaf R, Cuijpers P (2012). Parental loss by death during childhood: psychopathology in adulthood. Psychiatry Res.

