# Supplementary material for: Mental health of adolescents before and after the death of a parent or sibling
Source: Eur Child Adolesc Psychiatry. 2015 Mar 19;25(1):49–59. doi: 10.1007/s00787-015-0695-3 (PMC4698293; doi:10.1007/s00787-015-0695-3)
Supplement: Supplementary file 1 — Supplementary material 1 (DOCX 28 kb) [file 787_2015_695_MOESM1_ESM.docx]

Figure 1

Assessments used of family-bereaved participants

Pre-loss Post-loss

T1 T2

Group A

T2 T3

Group B

T3 T4

Group C

Figure 2

Assessments used of non-bereaved participants

Pre (-loss) Post (-loss)

T1 T2

Group A

T2 T3

Group B

T3 T4

Group C
